# Hypericin Induces Apoptosis in AGS Cell Line with No Significant Effect on Normal Cells

**DOI:** 10.22037/ijpr.2019.14904.12735

**Published:** 2020

**Authors:** Misagh Naderi, Mahtab Rahmani Cherati, Ali Mohammadian, Mohammad Baghery Bidhendy, Saeedeh Ghiasvand, Hadi Zare Marzouni, Hoda Aryan, Ehsan Jangholi, Mohammad Amin Javidi

**Affiliations:** a *Integrative Oncology Department, Breast Cancer Research Center, Motamed Cancer Institute, ACECR, Tehran, Iran. *; b *Department of Biology, Science and Research Branch, Islamic Azad University, Tehran, Iran. *; c *Department of Medical Biotechnology, Faculty of Medical Sciences, Tarbiat Modares University, Tehran, Iran. *; d *Young Researchers and Elite Club, Tehran Medical Sciences Branch, Islamic Azad University, Tehran, Iran. *; e *Clinical Research Development Center, Amir-almomenin Hospital, Tehran Medical Sciences Branch, Islamic Azad University, Tehran, Iran. *; f *Departments of Biology, Faculty of Science, Malayer University, Malayer, Iran. *; g *Student Research Committee, Mashhad University of Medical Sciences, Mashhad, Iran. *; h *Medical Students’ Scientific Association (MSSA), Tehran Medical Sciences Branch, Islamic Azad University Tehran, Iran. *; i *Clinical Research Development Center, Amir-almomenin Hospital, Tehran Medical Sciences Branch, Islamic Azad University, Tehran, Iran.*

**Keywords:** Hypericin, Herbal medicine, Gastric cancer, Anticancer drug, AGS, Apoptosis

## Abstract

It is of great importance to find an effective approach that not only eliminates gastric cancer cells but also do exhibits significant side effect to normal cells. Some studies have shown the effectiveness of hypericin against cancer cells. In this study, we evaluated the anti-cancer effect of Hypericin in the treatment of gastric cancer. In this study, the AGS cell line was exposed to different concentrations of hypericin for 24 and 48 h. Evaluation of cell death was done by MTT assay. The rate of apoptosis was measured by flow cytometry assay using Annexin V/ Propidium Iodide. The expression rate of *Bcl2*,* p53 *and *Bax* genes was evaluated by Real-time PCR test, and immunocytochemistry (*ICC*) analysis and western blotting was used for further evaluation of *p53*. MTT assay test showed that hyepricin induces 50% cell death in the concentration of 1 (µg/mL) and 0.5 (µg/mL) at 24 h and 48 h post-treatment, respectively, however no similar effect seen on fibroblast cells. Annexin/PI test revealed that cell apoptosis after exposure to hypericin for 24 h was 74%. Real-time PCR showed that expression level of *Bax*, *p53* and Bax genes increases and *Bcl2* gene decreases in AGS cell lines after treatment by hypericin. *ICC* analysis and western blotting for *p53* confirmed these data. The results of this study indicated that hypericin has the potential to be introduced as an effective treatment for gastric cancer. Therefore, it seems that this substance has potential to be utilized as anti-cancer drug.

## Introduction

Gastric cancer is one of the most common types of cancers and is ranked third among the most lethal cancers, with thousands of deaths per year occurring worldwide (1-3). The etiology of gastric cancer remains largly unknown, but several factors have been shown to contribute to the development of this cancer, including infections such as *Helicobacter Pylori* and *Epstein-Barr* virus. In addition, high salt intake, smoking, obesity and family history are also associated with gastric cancer risk (4-6).

Gastric cancer begins with uncontrolled proliferation of cells in the inner lining of the stomach. Primary symptoms of gastric cancer include heartburn, upper gastrointestinal pain, nausea and appetite loss. Other symptoms include weight loss, jaundice, difficulty swallowing and bloody stool (7).

Gastric cancer may spread to other parts of the body, including liver, lungs, bones and lymph nodes (8). In most cases, the disease is diagnosed in its advanced state and the initiation of treatment is delayed, and the survival rate is less than five years (3). Currently, treatments for this cancer include a combination of surgery, chemotherapy, and radiotherapy. However, these methods are possibly followed by irreparable complications for the patient. Furthermore, resistance of tumors to current treatments calls for development of novel anticancer drugs (9, 10). 

Today, the use of medicinal herbs in the treatment of many diseases is being considered by the scientific society(11-13). Hypericin is a naphthodianthrone extracted from *Hypericum* genus plant. In various studies, the photodynamic, anti-viral and anti-depressant effects of hypericin have been proved. Moreover, some studies have mentioned the cytotoxicity effect of hypericin. Hypericin cytotoxicity effect leads to inhibitory effects on the angiogenesis of cancer cells and induction of apoptosis (14-16).

Although studies have been done on the anticancer effects of Hypericin, the mechanism of action in which this product exerts its anti-cancer activity on gastric cancer cells is not well understood. Therefore, this study aimed to evaluate the anticancer effect of hypericin on AGS cell line and its effects on the expression level of important genes in apoptotic pathway.

## Experimental

*Materials and reagents*

AGS and normal fibroblast cell lines were purchased from Pasteur Institute of Iran. The cells were cultured in high glucose Dulbecco’s Modified Eagle’s Medium (HDMEM). Complete medium included HDMEM plus 10% fetal bovine serum, 100 mg/mL streptomycin and 100U/mL penicillin (all from Gibco, USA). 

Trypsin, Hypericin and MTT (3-(4, 5-Dimethyl-2-thiazolyl)-2,5-diphenyl-2-tetrazolium bromide) assay kit were obtained from Sigma Aldrich (USA). Immunocytochemistry (*ICC*) was performed using rabbit polyclonal anti-*p53* antibody, goat anti-rabbit IgG Fc (FITC) (Abcam, UK). PI (Abcam) was used to stain cell nuclei in *ICC*-test. All other reagents used in this study were obtained from Sigma Aldrich.

*Cell culture*

AGS and fibroblast cell lines were cultured in the complete medium and incubated at 37 °C with 5% CO_2_. The medium was changed every four days. When confluency of cells reached 90%, the cells were washed with PBS and then detached by trypsin. After detaching, trypsin was neutralized and cells were centrifuged at 1200 rpm, 37 °C for 5 min. These cells where then counted and utilized for tests.

*MTT assay and Apoptosis assay by flow cytometry*

For MTT assay in 24 and 48 h, 8000 or 6000 cells were seeded in each well of 96 well plates. After 24 h, culture media were replaced with fresh media containing various concentrations of hypericin in triplicate. After 24 or 48 h the media of each well was replaced with fresh media plus an appropriate amount of MTT solution based on manufacturer’s protocol. Purple formazan sediments appeared after 3 h. MTT containing media in the wells were depleted and 200 µL of DMSO was added to each well, in order to dissolve formazan crystals. The absorption of the solution in each well was determined by Biotek ELX800 microplate reader (BioTek Instruments, Inc.) at 570 nm.

The cellular death occurring after hypericin treatment was further investigated using Annexin V/PI apoptosis assay kit exactly based on manufacture’s manual. 

*Real-time PCR*

Total RNA was extracted by TRIzol reagent (Invitrogen) according to manufacturer’s recommendation. The quality and concentration of extracted RNA were determined by spectrophotometry. cDNA was synthesized using Takara (Japan) cDNA synthesis kit. The primers were designed to specifically amplify *Bax*, *Bcl2* and *p53* mRNAs (Table 1). GAPDH was used as reference gene. Real-time PCR was performed using Applied Biosystems 7500 Fast Real-Time PCR device and SYBR green master mix (Takara, Japan) with the following program: 1- Holding Stage: 95 °C/5 min 2- Cycling Stage: denaturing step: 95 °C/15 s, followed by annealing step 60 °C/30 s, amplification step 72 °C/20s (Number of Cycles: 40). 3- Melt curve analysis stage. The raw result were analyzed using 2^-^^∆∆Ct^ method to obtain fold change values.

*Immunocytochemistry*

AGS cells were cultured in 24 well plates. After treatment by hypericin, cells were fixed with 4% paraformaldehyde (10 min at 25 °C). The cells were then incubated at 4 °C with anti-*p53* antibody overnight. Cells were washed three times with PBS and incubated with FITC for 1 hour. Nuclei of cells were stained by DAPI and cells stained without primary antibody were employed as a negative control. 

*Western blotting*

*p53* expression at the protein level in normal untreated AGS cells and AGS cells treated with IC50 of hypericin was determined by western blotting. Cells were lysed and the same amount of protein from each sample was utilized for western blotting. Proteins were separated by 10% SDS-PAGE and transferred to nitrocellulose membranes. Then the membranes were incubated 1 hour with 5% nonfat milk at room temperature. Rabbit monoclonal antibody against *p53* protein (abcam) was utilized as the primary antibody. Membranes were incubated with primary antibody at 4 °C overnight. Rabbit monoclonal antibody against GAPDH was used as the control. Washing buffer contained Tris buffered saline and Tween 20. After washing step, the membranes were incubated with the secondary antibodiy goat Anti-Rabbit IgG H&L (HRP) (abcam). The complexes were visualized with an Immobilon Western Chemiluminescent HRP substrate (Millipore, USA). For quantification of the bonds, Image J (Fiji) software was used. Bands of GAPDH protein from each sample was used for normalization.

*Statistical analysis*

All statistical analysis performed by GraphPad Prism software (version 5.00) with *p *≤ 0.05 as significant level (error bars represent mean ± SD). To investigate whether the difference between groups were significant, *t*-test performed.

## Results

*Morphological change*

**Figure 1:**
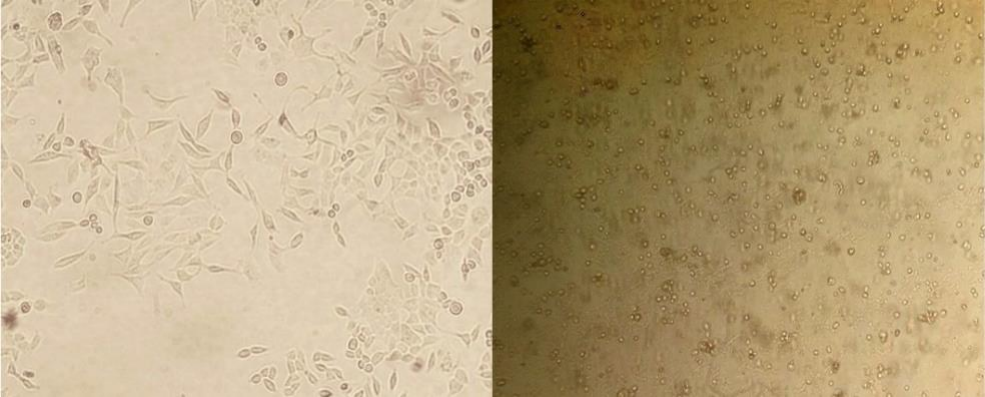
Morphological changes in AGS cells after treatment with IC50 dose of Hypericin for 24 h. Left, untreated AGS cells; right, exactly same cells after 24 h treatment by Hypericin (magnification 100X).

Treatment of AGS cells with hypericin caused cell morphology change and elongated cells were replaced by round cells as seen through invert microscope (Figure 1). This shape change may indicate apoptosis.

*MTT assay*

**Figure 2:**
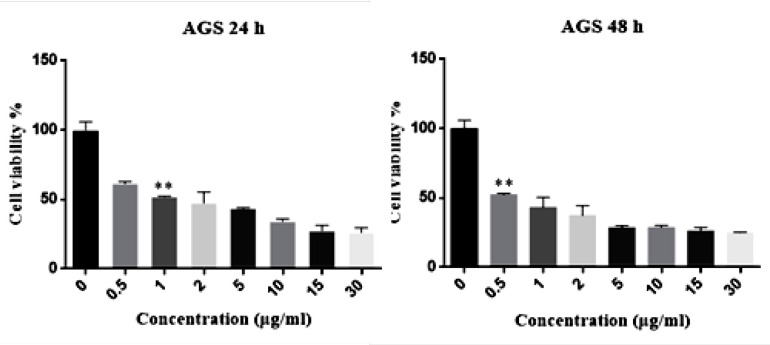
MTT assay results. The left diagram shows that the IC50 dose of Hypericin in 24 h was 1 (μg/mL) (*p*-value = 0.0074) and the right diagram reveals that IC50 dose of Hypericin in 48 h was 0.5 (μg/mL) (*p*-value = 0.0046).

**Figure 3:**
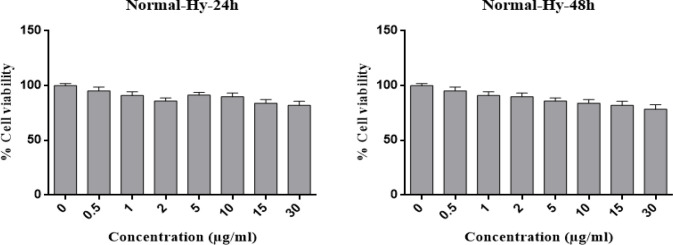
MTT assay results for the treatment of fibroblast normal cells with different concentrations of Hypericin for 24 h (left diagram) and 48 h (right diagram). Hypericin does not have a significant effect on fibroblast normal cells in concentrations up to 30 (μg/mL) in 24 and 48 h (*p* value= 0.05).

The IC50 of Hypericin on AGS cells was determined to be 1 μg/mL and 0.05 μg/mL in 24 and 48 h, respectively (Figure 2). Interestingly, even 30 μg/mL of hypericin did not have any significant effect on survival of normal fibroblast cells in 24 and 48 h (Figure 3).

*Flowcytomemtry Annexin V/PI-test*

**Figure 4:**
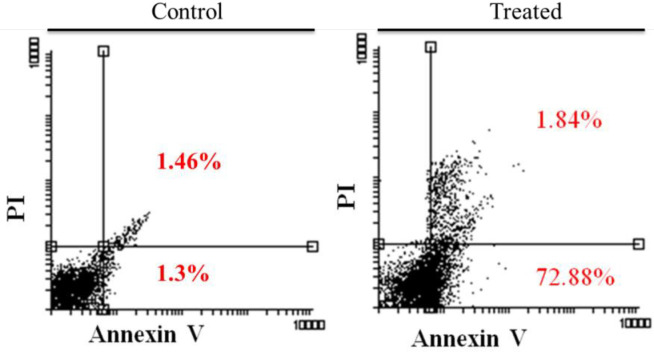
Annexin/PI flow cytometry results. Left diagram is the untreated AGS cells, and the right diagram is AGS cells which treated with 5 (μg/mL) of Hypericin for 24 h. After treatment, about 74 % of cells underwent apoptosis (right diagram, up-right and down-right quadrants) compared with untreated cells

On hypericin treatment AGS cells underwent apoptosis, as revealed by Annexin/PI flow cytometry (Figure 4). Treatment of cells with 5 μg/mL hypericin for 24 h, induced about 74% apoptosis and no necrosis (Figure 4).

*Real time PCR and ICC*

**Figure 5:**
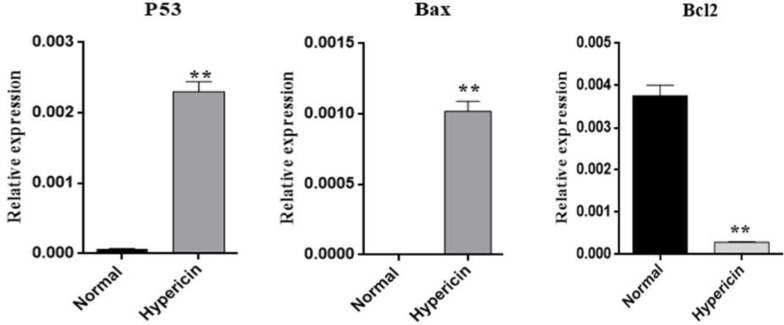
Real-time PCR results for investigating the change in relative expression of proapoptotic *Bax* and p53 genes and antiapoptotic gene *Bcl2*, after treatment of AGS cells with IC50 dose of Hypericin for 24 h. Hypericin induced overexpression of *p53* (*p*-value = 0.00451) and *Bax* genes (*p*-value = 0.0084) (left and center plots) and downregulated *Bcl2* (*p*-value = 0.0061) mRNA expression level (right plot) (Normal; untreated AGS cells).

**Figure 6:**
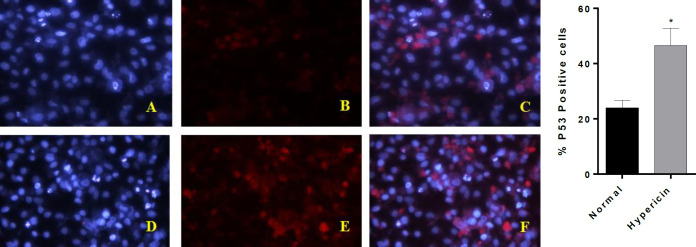
*ICC*-test for p53 protein. (A-C) are AGS untreated cells, (D-F) are AGS cells which treated with IC50 dose of Hypericin for 24 h. (A and D) are cells nuclei that stained with DAPI. B and E stained with anti p53 antibody-secondary antibody-FITC (C) is the merged picture of (A and B), F is the merged picture of (D and E). The diagram demonstrates quantification of *p53* positive cells (*p*-value = 0.0447). After treatment of AGS cells, red dots increased (E and F); which demonstrates about twofold *p53* upregulation at the protein level

To further investigate whether hypericin treatment induces apoptosis, the expression level of proapoptotic and antiapoptotic genes at mRNA level was studied. After treatment of AGS cells with the IC50 dose of hypericin for 24 h, the expression level of *Bax* and *p53* proapoptotic genes was increased, and the expression level of anti-apoptotic *Bcl2* was decreased (Figure 5). *ICC* confirmed that treatment of AGS cells with the IC50 dose of hypericin for 24 h causes *p53 *protein overexpression (Figure 6). 

*Western blot*

**Figure 7:**
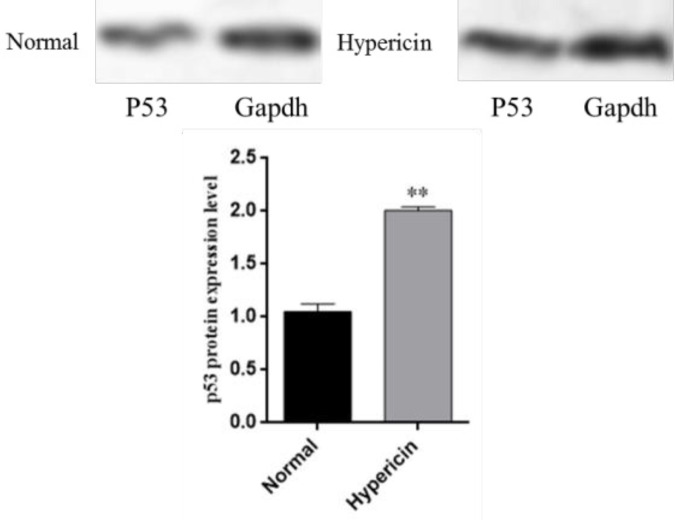
Western blot analysis for p53 protein after treatment of AGS cells with Hypericin. After treatment of AGS cells with the IC50 of Hypericin, p53 protein level increased about 2 fold more in treated cells (*p* value = 0.0071)

Western blot results revealed that after treatment of AGS cells with the IC50 dose of hypericin, *p53* is over-expressed at protein level (Figure 7). Western blot data confirmed that this treatment triggers upregulation of *p53* proapoptotic gene, which would in turn results in cell cycle arrest and apoptosis in treated cells (this phenomenon was demonstrated with Annexin *V/PI*-test either).

**Table 1:** Primers used for real time PCR

**Gene**	**Forward primer**	**Reverse primer**	**Amplicon length**
GAPDH	AAGTTCAACGGCACAGTCAAGG	CATACTCAGCACCAGCATCACC	121 bp
*p53*	TCCTCAGCATCTTATCCGAGTG	AGGACAGGCACAAACACGCACC	265 bp
*Bax*	CCAAGAAGCTGAGCGAGTGT	CCCAGTTGAAGTTGCCGTCT	156 bp
*Bcl-2*	TCTTTGAGTTCGGTGGGGTC	GTTCCACAAAGGCATCCCAG	153 bp

## Discussion

Gastric cancer is a common cancer with high mortality rate (17). The treatment of gastric cancer is done by surgery, radiotherapy, and chemotherapy. However, tumor resistance to radiotherapy and chemotherapy calls for development of novel anticancer drugs (9, 10, 18). *Hypericum perforatum* is a medicinal plant, having hypericin as one of its active ingredients. Previous studies have shown that hypericin has antioxidant effects and is cytotoxic to some cancer cell lines (17, 19). 

In a study by Mirmalek *et al.* on the effect of Hypericin on breast cancer cell line, they concluded that hypericin played a dose-dependent apoptotic impact on MCF-7 cell line. They showed that the IC50 hypericin and cisplatin on the MCF-7 cell line were 5 μg/mL and 20 μg/mL, respectively, indicating the effectiveness of hypericin at much lower concentrations compared with cisplatin. Moreover, they concluded that hypericin had a suitable cytotoxic effect on the breast cancer cell line and is a good candidate to be used in the treatment of breast cancer (17). Hamilton* et al.* showed that hypericin induced apoptosis in hypophysis adenoma cells AtT-20 and GH4C1 at 100 nM. Interestingly, a concentration of 10 μM did not induce apoptosis in human fibroblasts (20). Moreover, Kim *et al.* (21) demonstrated that that concentration of hypericin for growth inhibition in 50% of histiocytic lymphoma cells was 0.2 µM. Yi *et al*. (22) found that photoactivated hypericin at a concentration of 50 nM induced apoptosis through elevation of *Bax*-to *Bcl-2* ratio in RINm5F insulinoma cells. In the study caspase-3 and caspase-9 were also found to be elevated. In addition to inducing apoptosis, hypericin has also been reported to eliminate von-Hippel Lindau protein from cancerous cells and thus prevented growth of cancer cells (23). In contrast to this study and other reports on the apoptotic cell death caused by hypericin, Mikeš *et al*. (24) found that necrosis is the main type of cell death in human colon adenocarcinoma HT-29 cells treated with photodynamic therapy with 60-400 nM hypericin. Another report in which did not confirm our study was done by Do *et al.* (25). They found that hypericin, at a concentration of 0.5 μM, inhibited methylglyoxal-induced apoptosis. The action was mediated through changes in the expression level of *Bcl-2 *and *Bax*. 

We observed down-regulation of *Bcl-2* and up-regulation of Bax. Photoactivated hypericin (0.021 μM) in presence of genistein also down-regulated *Bcl-2* and up-regulated Bax in human breast adenocarcinoma cell lines MCF-7 and MDA-MB-231 (26). In a study by You *et al. *(27) the extract of *Hypericom perforatum, *the source of hypericin*,* also increased the expression of Bax and decreased the expression of *Bcl-2*.

Hypericin may physically interact with *Bcl2* family proteins (28)). In U87 MG and HCAEC cells, hypericin (500 nM) signiﬁcantly changed the distribution of *Bcl2* and *Bax* proteins in the dark (29). Hypericin also alters the distribution of *Bcl-2* in U-87 MG glioma cells (30) and in human coronary aorta endothelial cells (31). In the study by Chan *et al.* (32), six hour post hypericin photodynamic therapy, apoptotic nuclei were seen in HK-1 nasopharyngeal carcinoma cells. *Bax* translocation and formation of *Bax* channels in mitochondria membrane were mentioned as the causes of cell death in the study. As found by Balogová (30) apoptotic stimulus by hypericin photodynamic action on U-87 MG glioma cell line caused Bax translocation into mitochondria. In sonodynamic therapy with hypericin (25 μg/mL) in THP-1 macrophages *Bcl-2* protein was found to be down-regulated (33). 

In a survey by Acar *et al.* (34) it was shown that treatment of MCF-7 breast cancer cells with hypericin (7.5, 5 and 1 μM ) did not alter expression of *p53* gene, although it did induce apoptosis. Furthermore, the level of *Bcl-2* was not altered in both *p53*-null and wt-*p53*-expressing HCT-116 cells after hypericin photodynamic therapy, which is in contrast to our findings (35). 

Xu *et al.*(36) found that hypericin (6.25-200 ng/mL) photodynamic therapy induced apoptosis and G2/M phase cell cycle arrest in Adult T-cell leukemia. *Bcl-2* was down-regulated while Bax and p53 were up-regulated. Gamasaee *et al.* (37) found that* p53* was up-regulated in MDA-MB-175-VII cells which underwent apoptosis when treated with hypericin. Halaburková *et al.*(38) showed that the induction of apoptosis in colon cancer cells HT-29 and HCT 116 co-treated with the histone deacetylase inhibitor sodium phenylbutyrate+ hypericine  was *P53*-dependent. The authors also suggested that the effect was due to hypericin photodynamic therapy rather than sodium phenylbutyrate. These results are also in line with our findings. However, the results by Lee *et al.* suggested that hypericin (0.06-1 µ/mL) mediated phototherapy acted independently from p53, and the authors suggested that photodynamic therapy may have equal efficacy regardless of the presence or absence of *p53* (39). In a similar report Weller *et al.* (40) had found that in human malignant glioma cells, hypericin induced apoptosis independently of *p53*.

Finally we suggest that the synergistic effect of YM155 (29) or genistein (26) with hypericin may be also tested in induction of apoptosis in AGS cells.

## Conclusion

The results of this study indicated that hypericin is a cytotoxic and apoptosis-inducing substance on AGS cell line. Hypericin showed low IC50 in AGS gastric cancer cell line but was well tolerated by fibroblasts at even higher concentrations. These results suggest hypericin is potentially be proposed as a suitable treatment for gastric cancer. We propose that the anticancer effects of hypericin be studied through *in*-*vivo* studies.
